# Association of Irisin with Fat Mass, Resting Energy Expenditure, and Daily Activity in Conditions of Extreme Body Mass Index

**DOI:** 10.1155/2014/857270

**Published:** 2014-04-22

**Authors:** María Pardo, Ana B. Crujeiras, María Amil, Zaida Aguera, Susana Jiménez-Murcia, Rosa Baños, Cristina Botella, Rafael de la Torre, Xavier Estivill, Ana B. Fagundo, Jose M. Fernández-Real, José C. Fernández-García, Gema Fruhbeck, Javier Gómez-Ambrosi, Roser Rodríguez, Francisco J. Tinahones, Fernando Fernández-Aranda, Felipe F. Casanueva

**Affiliations:** ^1^Laboratory of Molecular and Cellular Endocrinology, Health Research Institute of Santiago (IDIS), University Hospital of Santiago (XXIS/SERGAS) and Santiago de Compostela University (USC), 15706 Santiago de Compostela, Spain; ^2^CIBER Fisiopatología de la Obesidad y la Nutrición (CIBERobn), 15706 Santiago de Compostela, Spain; ^3^Obesidómica Group, Laboratory 3, Health Research Institute of Santiago (IDIS), University Hospital of Santiago (XXIS/SERGAS), 15706 Santiago de Compostela, Spain; ^4^Cancer Epigenetics and Biology Program (PEBC), Bellvitge Biomedical Research Institute (IDIBELL), 08908 Barcelona, Spain; ^5^Department of Psychiatry, University Hospital of Bellvitge-IDIBELL, 08907 Barcelona, Spain; ^6^Department of Clinical Sciences, School of Medicine, University of Barcelona, 08007 Barcelona, Spain; ^7^Department of Psychological, Personality, Evaluation and Treatment of the University of Valencia, 46010 Valencia, Spain; ^8^Department of Basic Psychology, Clinic and Psychobiology of the Jaume I University, Castelló, 12071 Castelló de la Plana, Spain; ^9^Human Pharmacology and Clinical Neurosciences Research Group, Neuroscience Research Program, Hospital del Mar Medical Research Institute (IMIM), Barcelona, Spain; ^10^Center for Genomic Regulation (CRG), 08003 Barcelona, Spain; ^11^Centro de Investigación Biomédica en Red en Epidemiología y Salud Pública (CIBERESP), 08036 Barcelona, Spain; ^12^Department of Diabetes, Endocrinology and Nutrition, Institut d'Investigació Biomèdica de Girona (IdlBGi),Hospital Dr Josep Trueta, Girona, Spain; ^13^Department of Diabetes, Endocrinology and Nutrition, Hospital Cliínico Universitario Virgen de Victoria, 29010 Maálaga, Spain; ^14^Department of Endocrinology and Nutrition, University Clinic de Navarra, University of Navarra, 31008 Pamplona, Spain

## Abstract

FNDC5/irisin has been recently postulated as beneficial in the treatment of obesity and diabetes because it is induced in muscle by exercise, increasing energy expenditure. However, recent reports have shown that WAT also secretes irisin and that circulating irisin is elevated in obese subjects. The aim of this study was to evaluate irisin levels in conditions of extreme BMI and its correlation with basal metabolism and daily activity. The study involved 145 female patients, including 96 with extreme BMIs (30 anorexic (AN) and 66 obese (OB)) and 49 healthy normal weight (NW). The plasma irisin levels were significantly elevated in the OB patients compared with the AN and NW patients. Irisin also correlated positively with body weight, BMI, and fat mass. The OB patients exhibited the highest REE and higher daily physical activity compared with the AN patients but lower activity compared with the NW patients. The irisin levels were inversely correlated with daily physical activity and directly correlated with REE. Fat mass contributed to most of the variability of the irisin plasma levels independently of the other studied parameters.* Conclusion*. Irisin levels are influenced by energy expenditure independently of daily physical activity but fat mass is the main contributing factor.

## 1. Introduction

A balance between energy intake and energy expenditure is responsible for body weight maintenance; disturbances in this balance may cause individuals to be underweight, anorexic, overweight, or obese [1, 2]. Signals secreted by peripheral tissues, such as fat or the digestive tract, are active participants in body weight regulation by acting at central level [3]. Skeletal muscle has recently been shown to function as a peripheral endocrine organ by releasing peptide signal molecules known as myokines, which are implicated in the regulation of several physiological and metabolic pathways [4, 5]. Myokines secreted during muscle contraction may exert a protective role against illnesses associated with sedentary lifestyles [6]. The muscle-secreted peptide irisin has been recently described as a cleavage product of the type I membrane protein fibronectin type III domain containing 5 (FNDC5) [7]. In muscle, FNDC5 is induced by the peroxisome proliferator-activated receptor *γ* (PPAR*γ*) transcriptional coactivator PGC1-*α*, which is described as an essential mediator in the exercise-induced energy expenditure cascade and stimulates several beneficial effects of exercise in muscle tissue [7, 8]. After physical activity, FNDC5 has been reported to promote white adipose tissue browning by increasing thermogenesis and energy expenditure. Thus, augmentation of circulating irisin levels by forced liver expression of FNDC5 using adenoviral vectors improves glucose tolerance and reduces obesity of mice fed on a high-fat diet [7]. Under this context, irisin was rapidly postulated to be beneficial in the treatment of obesity, diabetes, and a wide range of pathological conditions characterized by a variable imbalance of energy demand and expenditure [4, 9–13].

Unexpectedly, some studies have indicated that the circulating irisin levels in humans correlate positively with parameters of adiposity such as BMI and are the highest in obese individuals [14–17]. On the other hand, a recent paper reports that irisin correlates negatively with BMI, waist-hip ratio, and fat mass in men and that circulating irisin is lower in nondiabetic overweight and obese men [18, 19]. Our group has established a new perspective for FNDC5 by describing that FNDC5/irisin is also an adipokine expressed and secreted by adipocytes from white adipose tissue (WAT) in rats and humans [16]. We have observed that short-term endurance exercise increases FNDC5/irisin secretion by adipose tissue, while food restriction decreased secretion. Accordingly, anorectic animals showed discrete secretion of FNDC5/irisin, while fat from obese animals oversecretes FNDC5/irisin. Thus, we postulated that adipose tissue may contribute to circulating FNDC5/irisin and that the contribution may vary depending on the physiological state or the pathological situation.

The objective of this study was to evaluate the circulating levels of irisin in conditions of extreme BMI, such as anorexia and obesity, and the correlations of irisin with basal metabolism and daily activity.

## 2. Materials and Methods

### 2.1. Subjects

A total of 145 females participated in this study after being informed about the research and signing informed consent. Thirty patients were anorexic (AN; BMI 17.3 ± 1.8 kg/m^2^; 28.1 ± 9.4 years), 66 were obese (OB; BMI 42.8 ± 6.7 kg/m^2^; 29.0 ± 6.2 years; 21.2% with type 2 diabetes mellitus), and 49 were healthy, normal-weight controls (NW; BMI 21.7 ± 1.6 kg/m^2^; 44.5 ± 11.4 years).

Enrolment into the study occurred between January 2010 and December 2012. All consecutive patients were referred to seven centers from six Spanish sites (all involved in the CIBERobn Spanish Research Network): the Eating Disorders Unit (Department of Psychiatry, University Hospital of Bellvitge-IDIBELL, Barcelona), the Department of Endocrinology at the University Hospital of Santiago (Santiago de Compostela), the Department of Diabetes, Endocrinology and Nutrition (Clinic University Hospital Virgen de Victoria, Málaga), the Department of Endocrinology (University of Navarra, Pamplona), the Diabetes, Endocrinology and Nutrition Department, Biomedical Research Institute of Girona (IdIBGi-Doctor Josep Trueta Hospital, Girona), the Hospital del Mar Research Institute (IMIM-Hospital del Mar, Barcelona), and the Department of Basic Psychology, Clinic and Psychobiology (University Jaume I, Castellón).

The AN patients were diagnosed by experienced clinicians (according to DSM-IV-TR diagnostic criteria) [20] using the structured clinical interview for DSM IV Axis I disorders (SCID-I) [21]. These AN patients at extreme BMI were hospitalized, hence showing weaken health and very limited physical activity.

The interviewers were trained for the administration of these instruments. The following exclusion criteria were applied to the clinical cases groups (AN and obese patients): (1) male; (2) age under 18 or over 60 years, and (3) a comorbid binge eating disorder in the obese patients (according to DSM-IV-TR criteria, using SCID-I) [22]. Healthy controls were recruited by several methods including word-of-mouth recruitment and advertisements in the local university. Prior to assessment, the healthy controls were asked about lifetime or current presence of an endocrine disease or obesity. The lifetime history of health or mental illnesses profile was based on the general health questionnaire (GHQ)-28 [23]. The following exclusion criteria were applied to the healthy control groups (NW): (1) individuals who have suffered a lifetime eating disorder (assessed using SCID-I), (2) age under 18 and over 60 years, (3) lifetime obesity (BMI > 30), and (4) being male.

The procedures were approved by the ethical committees of each of the aforementioned institutions.

### 2.2. Anthropometric, Body Composition, and Resting Energy Expenditure Measurements

Anthropometric, body composition, and resting energy expenditure (REE) measurements were performed by bioelectrical impedance (TANITA MC-180 Multifrequency; TANITA Corporation of America, Inc, Arlington Heights, IL, USA), equipped with 8 tactile electrodes with a platform with 2 electrodes for each foot and 2 handgrips with 2 electrodes each. The bioelectrical impedance analysis was chosen since it is able to predict the REE without bias with respect to more costly equipment and invasive procedures needed to directly measure REE [24].

### 2.3. Activity Variable

Physical activity was evaluated with the Actiwatch AW7 (Actiwatch AW7; CamNtech Ltd., Cambridge Neurotechnology, Cambridge, UK), which is a small (39 × 32 × 9) and light-weight (10.5 g) accelerometer that measures sleep-wake physical activity. The Actiwatch is worn on the nondominant wrist for 6 days (4 week days and 1 weekend), from 00:00 hr on day 1 to 00:00 hr on day 7. The data were collected over a 1-minute epoch length, and a 6-day average was calculated. The analysis was performed using the Actiwatch 7 software (CamNtech Ltd. and Cambridge Neurotechnology Ltd., Cambridge, UK). The Actiwatch AW7 originates from the Actiwatch AW4, which has similar reliability to other accelerometers [25] and is an adequate instrument for assessing daily physical activity [26].

### 2.4. Measurement of Plasma Irisin and Other Biochemical Variables

The quantitative measurement of irisin in human plasma samples was performed using a commercial enzyme-linked immunosorbent assay (ELISA) kit directed against amino acids 31–143 of the FNDC5 protein (Irisin ELISA Kit EK-067-52; Phoenix Pharmaceuticals Inc., CA) according to the manufacturer's instructions. The absorbance from each sample was measured in duplicate using a spectrophotometric microplate reader at wavelength of 450 nm (Versamax Microplate Reader; Associates of Cape Cod Incorporated, East Falmouth, MA).

Insulin was analysed by an immunoradiometric assay (BioSource International, Camarillo, CA, USA) in a Beckman Coulter (Fullerton, CA, USA), showing 0.3% cross-reaction with proinsulin. The intra- and interassay CV were 1.9% and 6.3%, respectively. Glucose was measured using a Dimension Autoanalyzer (Dade Behrng, Deerfield, IL, USA). The homeostatic model assessment index (HOMA-IR) was calculated following the formula (fasting plasma glucose (mg/mL) × fasting plasma insulin (mU/L)/405), as described elsewhere [27].

### 2.5. Adipose Tissue Biopsies and FNDC5/Irisin Immunodetection

The human adipose tissue specimen was obtained with written informed consent approved for this particular study by the Comité Ético de Investigación Clínica de Galicia—CEIC de Galicia (Spain) according to the Declaration of Helsinki. Adipose tissue was obtained from NW (body mass index < 35) who underwent cholecystectomy surgery and OB patients (body mass index > 35) who underwent laparoscopic gastrectomy surgery. The visceral fat was located in the hypogastric region around the internal organs, and the subcutaneous fat was located in the mesogastric region. The tissues were transported from the operating room to the laboratory in sterile KRH buffer with penicillin (100 U/mL) and streptomycin (100 *μ*g/mL). Secretome and tissues were collected and processed for immunodetection as previously described [5, 16].

### 2.6. Statistical Analysis

The sample size of the current trial was estimated by calculating the differences in irisin between the OB and NW patients according to the equation reported by Mera et al. [28]. The sample size was established at a minimum of 26 volunteers per group to detect differences according to the adiposity levels.

The normal distribution of the variables was tested using the Kolmogorov-Smirnov and the Shapiro-Wilk tests. The irisin levels exhibited a nonparametric distribution. However, based on the sample size of >60 subjects included in the current study, a one-way ANOVA was used to study the differences between AN, NW, and OB subjects adjusted by age. The potential association between anthropometric and biochemical parameters with and irisin levels was evaluated using the Pearson coefficient test. Multivariate linear regression models were fitted to explain the variations in the irisin plasma levels. Data are reported as the mean ± SE, and confidence intervals (95% CI) are used to describe the linear coefficient (B) values.

The statistical analysis was performed using SPSS version 15.0 software (SPSS Inc., Chicago, IL) for Windows XP (Microsoft, Redmond, WA). *P* ≤ 0.05 was considered to be statistically significant.

## 3. Results

**Figure 1:**
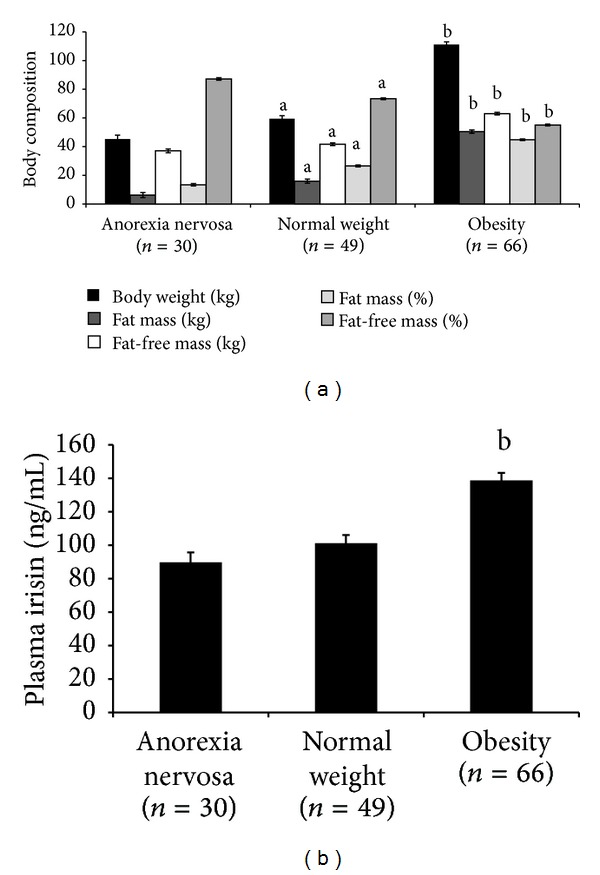
Body composition and plasma irisin circulating levels. Body weight (kg), fat mass (kg), fat-free mass (kg), fat mass (%), and fat-free mass (%) are shown in the anorexia nervosa, normal weight, and obese groups. (a) Plasma irisin concentration in the three experimental groups (b). The data are shown as the mean (SE). Statistically significant differences are denoted as (a) *P* < 0.001 versus anorexia nervosa and (b) *P* < 0.001 versus normal weight and anorexia nervosa.

As expected, the OB subjects exhibited higher body weight with higher fat mass and fat-free mass compared with the AN and NW subjects, with the differences being statistically significant after adjusting by age (Figure 1(a)). The NW subjects exhibited statistically higher body weight compared with the AN subjects accompanied by higher fat mass and fat-free mass. Considering the body composition as a percentage of the total body weight, the AN subjects exhibited higher fat-free mass than the NW and OB subjects (Figure 1(a)).

The differences in the anthropometrical parameters were consistent with the significantly increased plasma irisin concentrations in the OB subjects compared with the AN and NW groups (Figure 1(b)). The NW subjects exhibited a slight but not statistically significant increase compared with the AN subjects (*P* > 0.05).

**Figure 2:**
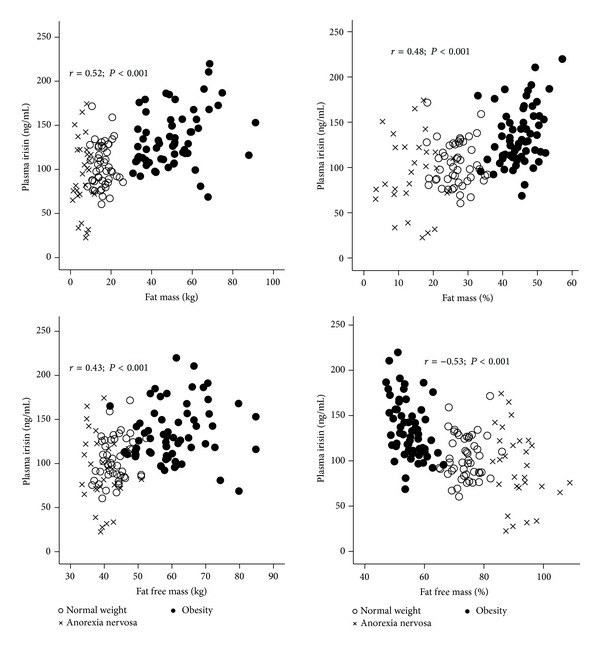
Correlation of circulating irisin with fat mass and fat-free mass. Plasma irisin correlation with fat mass (kg), fat mass (%), fat-free mass (kg), and fat-free mass (%) for the three groups (NW, AN, and OB) is shown.

Further analysis showed that the irisin levels were positively correlated with two parameters of adiposity (i.e., body weight (*r* = 0.52; *P* < 0.001) and BMI (*r* = 0.52; *P* < 0.001)). The irisin plasma levels were not correlated with height (*r* = −0.05; *P* > 0.05). The correlations indicated that irisin strongly reflects the body fat mass component, which is further supported by the bioelectrical impedance analysis of body composition in which the irisin plasma levels correlated with the fat mass measured in kg (*r* = 0.52; *P* = 0.001; Figure 2) and the percentage of fat mass (*r* = 0.48; *P* < 0.001). However, the muscle component may also contribute to irisin circulating levels as shown by the positive correlation between irisin and fat-free mass (*r* = 0.43; *P* = 0.001; Figure 2). An inverse correlation was observed between the irisin levels and the percentage of fat-free mass (*r* = −0.53; *P* < 0.001). The associations between irisin and fat mass and fat-free mass were maintained after adjusting for the studied groups (data not shown). In addition, a direct association was observed between plasmatic levels of irisin and glucose (*r* = 0.22; *P* = 0.0026), insulin (*r* = 0.34: *P* < 0.001), and HOMA-IR (*r* = 0.33; *P* = 0.001). This positive association was maintained especially for insulin and HOMA-IR after adjusting for the studied groups.

**Figure 3:**
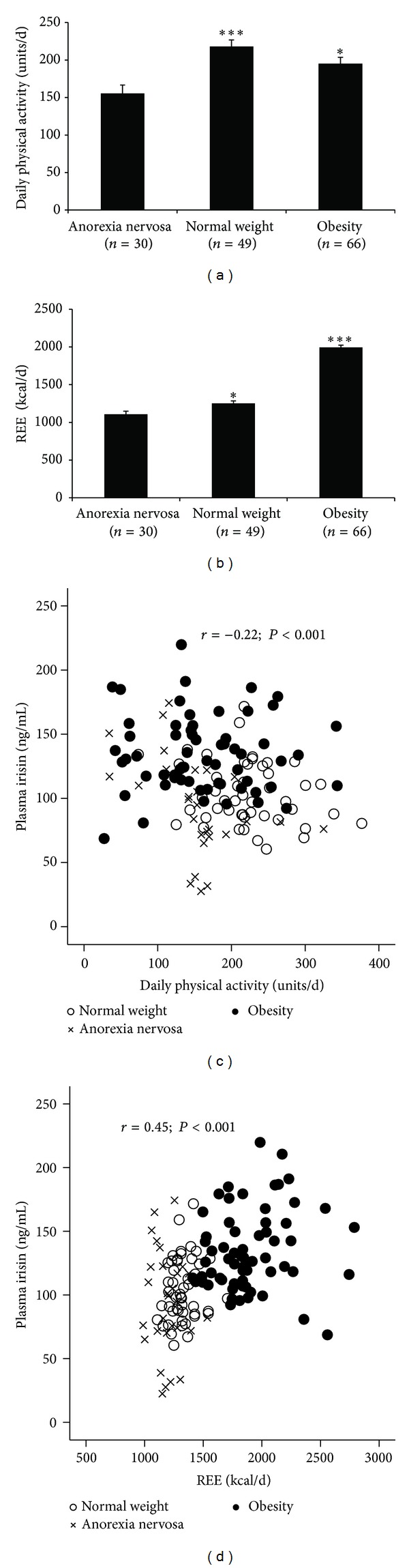
Daily physical activity and resting energy expenditure and its correlation with irisin. The average daily physical activity (units/day) and resting energy expenditure (REE: kcal/day) for the AN, NW, and OB subjects are shown in (a) and (b), respectively. Asterisk (_ _*P < 0.05 and _ _***P < 0.001) denotes statistically significant differences between normal weight and anorexia nervosa or obesity versus anorexia nervosa and normal weight. The correlations between the circulating irisin and the daily physical activity and REE for the three groups are presented in (c) and (d).

Because irisin secretion was shown to be induced by exercise and energy expenditure [7], the daily physical activity and resting energy expenditure (REE) were evaluated in the three BMI groups. The Actiwatch analysis in the OB subjects exhibited statistically lower daily physical activity with higher resting energy expenditure (REE) compared with the NW subjects adjusted by age (Figures 3(a) and 3(b), resp.). Therefore, the OB subjects exhibited higher daily physical activity compared with the extreme AN hospitalized subjects displaying very limited activity but lower activity compared with the NW subjects (*P* < 0.05). Obesity was accompanied by increased REE compared with the AN and NW subjects (*P* < 0.05). The plasma irisin levels were inversely correlated with daily physical activity (*r* = −0.22; *P* = 0.001) and directly correlated with REE (Figures 3(c) and 3(d)). After adjusting by the BMI group, only the association between irisin and REE was maintained (*r* = 0.34; *P* = 0.001), and the daily physical activity was not statistically significant (*r* = −0.06; *P* = 0.432).

To further investigate the potential factors involved in the changes in the irisin plasma levels, a multiple regression model was performed including REE and physical activity, adjusting for age, and BMI group (Table 1). The regression model predicting changes in irisin plasma levels showed a 21% (*P* < 0.001) of the irisin levels variability, mainly depending on REE, whereas physical activity was not statistically associated. When fat free mass was included in the regression model, the association between REE and irisin plasma levels persisted. However, when the same analysis was performed including fat mass in the multivariate model, REE becomes significantly unassociated with irisin circulating levels, whereas that fat mass was the main factor and explained approximately 30% (*P* < 0.05) of the irisin concentration variability independently of age, BMI group, fat-free mass, daily physical activity, and REE (Table 1). An increase in 1 kg of fat mass was associated with a 2-fold increase in the irisin plasma levels (*P* = 0.003). These results indicate that REE influence the irisin circulating levels but in a lesser extent than fat mass.

**Figure 4:**
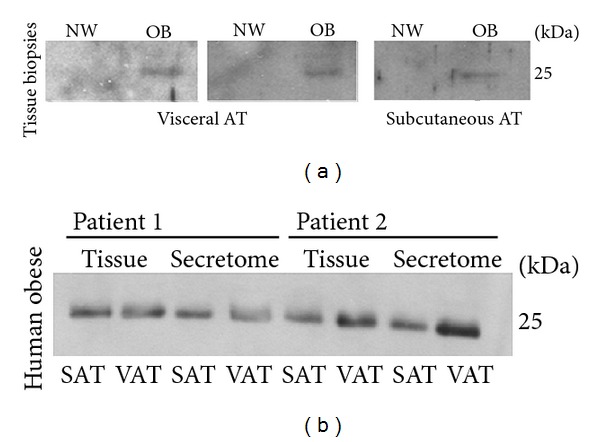
FNDC5/irisin detection on human adipose tissue. Representative immunoblot detection of FNDC5/irisin in human visceral adipose tissue (VAT) and subcutaneous adipose tissue (SAT) biopsies from NW and OB subjects (a); FNDC5/irisin immunodetection in human SAT and VAT biopsies and their secretome from OB individuals (b).

In order to assay the participation of adipose tissue on irisin circulating levels, the immunodetection of this protein was tested on adipose tissue biopsies from healthy and obese individuals. As shown in Figure 4, FNDC5/irisin was detected on visceral and subcutaneous adipose tissue secretion from NW and OB individuals (Figure 4(a)) and in OB at both intracellular and secretion level (Figure 4(b)).

## 4. Discussion 

Previous studies are rather controversial, and there is no general agreement about circulating irisin levels and their correlation with BMI [14, 15, 17, 18]. The current study performed with patients in conditions of extreme BMI confirms the positive correlation of circulating irisin and different parameters of adiposity including body weight, BMI, and fat mass. This work contributes to novel valuable information by showing that circulating irisin levels correlate positively with resting energy expenditure and negatively with daily physical activity. Therefore, the inverse correlation of irisin with daily physical activity and the direct relationship between irisin and adiposity indicate that adipose tissue may be a primary inducer of irisin secretion, especially in obesity.

The recent discovery of the PGC1-*α*-dependent myokine FNDC5/irisin, which mediates brown-fat development and thermogenesis in white fat, has opened a new field of research [4, 7, 9–13]. The soluble form of FNDC5 binds to unidentified receptors on the surface of WAT to induce the expression of Ucp1 and other BAT-associated genes partly via increased PPAR-*α* expression. As a result, irisin may act as a muscle-derived energy-expenditure signal that directly communicates with adipose tissue and induces browning. This effect may improve the WAT metabolic profile and enhance whole-body energy expenditure, making irisin a potential new target for the treatment of metabolic diseases.

The possible beneficial effect of irisin on the treatment of obesity has been challenged by recent reports showing unexpected high levels of irisin in obese animals and humans [14–17] as well as its association with the insulin resistance onset in association with weight regain [29]. Moreover, it was observed that in obese individuals, body weight and BMI reduction by bariatric surgery or by nutritional intervention significantly reduced irisin circulating levels [15, 17] and its depletion after an energy restricted program is associated with the reduction of important lipid [30] and carbohydrate [31] metabolism biomarkers in patients with metabolic syndrome. Other reports have described conflicting data indicating a lack of correlation between the circulating irisin concentration and the adiposity parameters [18]. This discrepancy could be due to methodological differences because there is no consensus concerning the identity of the soluble portion of the FNDC5 membrane protein, the mechanism of secretion, or the presence of variations on target epitopes among manufacturers. However, most published reports indicate correlations between irisin and adiposity levels. The present work further corroborates these findings; irisin plasma levels were elevated in obesity, and a strong relationship was found between irisin and several adiposity parameters.

In the current work, the obese subjects exhibited higher REE compared with the normal weight and anorexia nervosa subjects and it was directly related to irisin levels, independently of daily physical activity. This association between irisin and energy expenditure was previously reported in a cohort of postmenopausal women and it was proposed that this association could be explained by the irisin action on the brown adipose tissue thermogenesis [32]. However, the association between irisin and REE appears to be masked by the strong association between fat mass and irisin plasma levels. Fat mass was revealed as the main factor explaining approximately 30% of the variability in the irisin plasma levels independently of age, fat free mass, daily physical activity, REE, and BMI group.

The fact that daily physical activity was found to be independent of plasma irisin levels in our work may seem contrary to what was expected. Bostrom's results have shown the expression and secretion of irisin after endurance exercise [7], which cannot be compared to normal daily activity assayed in our paper. Moreover, there are studies that, in contrast to the initial report by Bostrom, have shown no variations of FNDC5 mRNA expression in muscle or at circulating level after aerobic or strength exercise training [15, 33, 34]. On the other hand, an increase of irisin circulating levels was observed after short-term exercise (sprints) which suggest that irisin secretion is dependent on acute or chronic sorts of exercise with a possible counter-regulation/adaptation over time [35]. Therefore, our results in relation to daily activity are coherent with previous evidences in the bibliography.

Our group has previously described that FNDC5/irisin is a myokine and an adipokine expressed and secreted by WAT [16]. Interestingly, we observed that explants of adipose tissue from obese animals or humans oversecreted irisin compared with the control subjects. Therefore, we suggest that the muscle/adipose secretion ratio may vary and may be affected by the physiological situation; during exercise, muscle tissue strongly participates in the FNDC5 circulating levels, while in atypical BMI cases, such as obesity, the adipose tissue would actively increase the circulating FNDC5/irisin. The current work further reinforces this hypothesis, because the main difference between the obesity and normal weight and anorexia nervosa subjects is the higher fat mass; therefore, adipose tissue could be involved in the circulating irisin concentration differences observed depending on the BMI as reinforced in preliminary experiments assaying FNDC5/irisin immunodetection in adipose tissue from NW and OB individuals in Figure 4.

The results in the present work with previous reports [14–17, 36, 37] might suggest a possible mechanism of resistance to irisin in situations of elevated BMI as previously observed in other adipokines (e.g., leptin) [38]. The elevated concentration of circulating irisin in overweight and obese subjects suggests that a great amount of BAT would be expected in these patients. Although it is now clear that BAT is present and functional in adult humans, significant low amounts of BAT have been described in overweight and obesity, which supports the irisin-resistance hypothesis [39, 40]. However, further research should be performed to confirm this hypothesis.

In conclusion, the current work demonstrates that irisin circulating levels is associated with energy expenditure independently of daily physical activity but in a lesser degree than fat mass, which was revealed as the main factor influencing the circulating irisin concentration in obese women with extreme BMI from anorexia nervosa to obesity. Therefore, these findings reinforce the positive relationship between circulating irisin and adiposity and the relevance of WAT-secreted irisin in situations of elevated BMI, such as obesity. Further studies are needed to elucidate whether irisin is involved in obesity development or irisin resistance that exist in an obesity state to counteract the metabolic disturbances related to excess adiposity.

## Figures and Tables

**Table 1:** Independent effects of body composition and activity markers on variations in irisin plasma levels.

	*B* (95% CI)	*P* value
Model 1*		
REE (Kcal/d)	0.35 (0.01; 0.005)	0.001
Daily physical activity (units/d)	−0.14 (−0.17; 0.01)	0.088
*Corrected R* ^2^ = 0.21		*<0.001 *
Model 2*		
REE (Kcal/d)	2.15 (0.06; 0.33)	0.006
Daily physical activity (units/d)	−0.10 (−0.15; 0.03)	0.202
Fat-free mass (Kg)	−1.81 (−9.81; −0.85)	0.020
*Corrected R* ^2^ = 0.23		*<0.001 *
Model 3*		
REE (Kcal/d)	−0.43 (−0.08; 0.01)	0.060
Daily physical activity (units/d)	−0.12 (−0.16; 0.02)	0.139
Fat mass (Kg)	0.94 (0.74; 2.31)	<0.001
*Corrected R* ^2^ = 0.28		*<0.001 *
Model 4*		
REE (Kcal/d)	0.09 (−0.32; 0.14)	0.435
Daily physical activity (units/d)	0.07 (−10.17; 9.09)	0.119
Fat mass (Kg)	1.7 (0.6; 2.8)	0.003
Fat-free mass (Kg)	1.3 (−4.8; 7.5)	0.657
*Corrected R* ^2^ = 0.28		*<0.001 *

*Adjusted for age and BMI group (0: anorexia nervosa; 1: normal weight; 2: obesity).
